# Supplementary figures and images for: Aspalathus linearis suppresses cell survival and proliferation of enzalutamide-resistant prostate cancer cells via inhibition of c-Myc and stability of androgen receptor
Source: PLoS One. 2022 Jul 1;17(7):e0270803. doi: 10.1371/journal.pone.0270803 (PMC9249401; doi:10.1371/journal.pone.0270803)

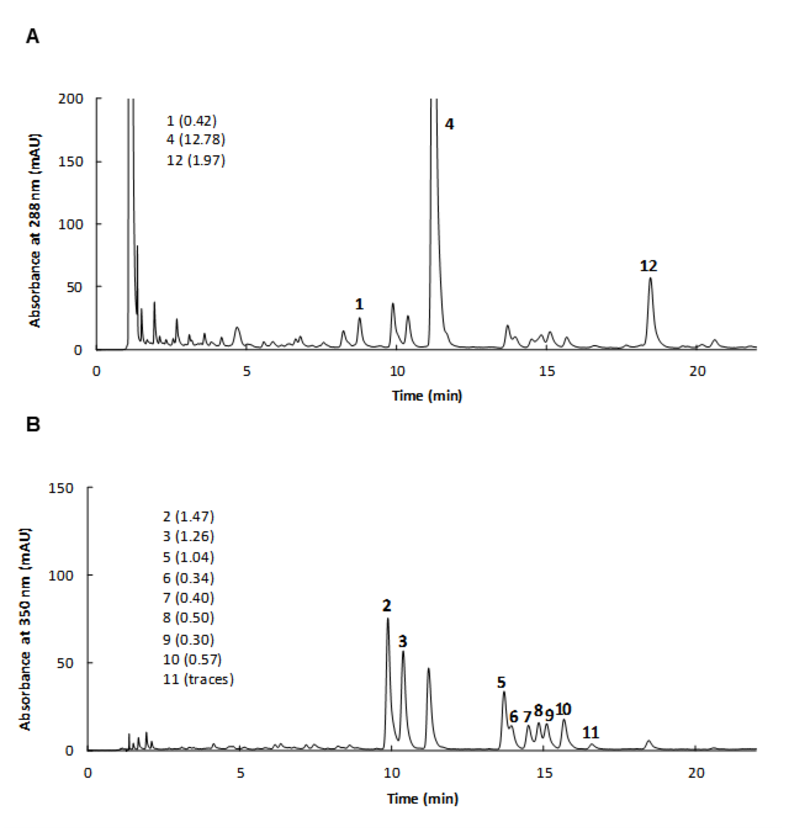

Supplement: S1 Fig — The GRT is the same production batch lot used in our previous study [7] and this figure is the same as Fig 1 in [7]. The components of GRT was analyzed at (A) 288 and (B) 350 nm. The content value of each numbered compounds (1, PPAG; 2, isoorientin; 3, orientin; 4, aspalathin; 5, bioquercetin; 6, vitexin; 7, hyperoside; 8, rutin; 9, isovitexin; 10, isoquercitrin; 11, luteoloside; 12, nothofagin) is indicated in brackets after the number on the chromatograms. Component is expressed as g/100 g GRT. (TIF) [file pone.0270803.s001.tif]

## Slide 1
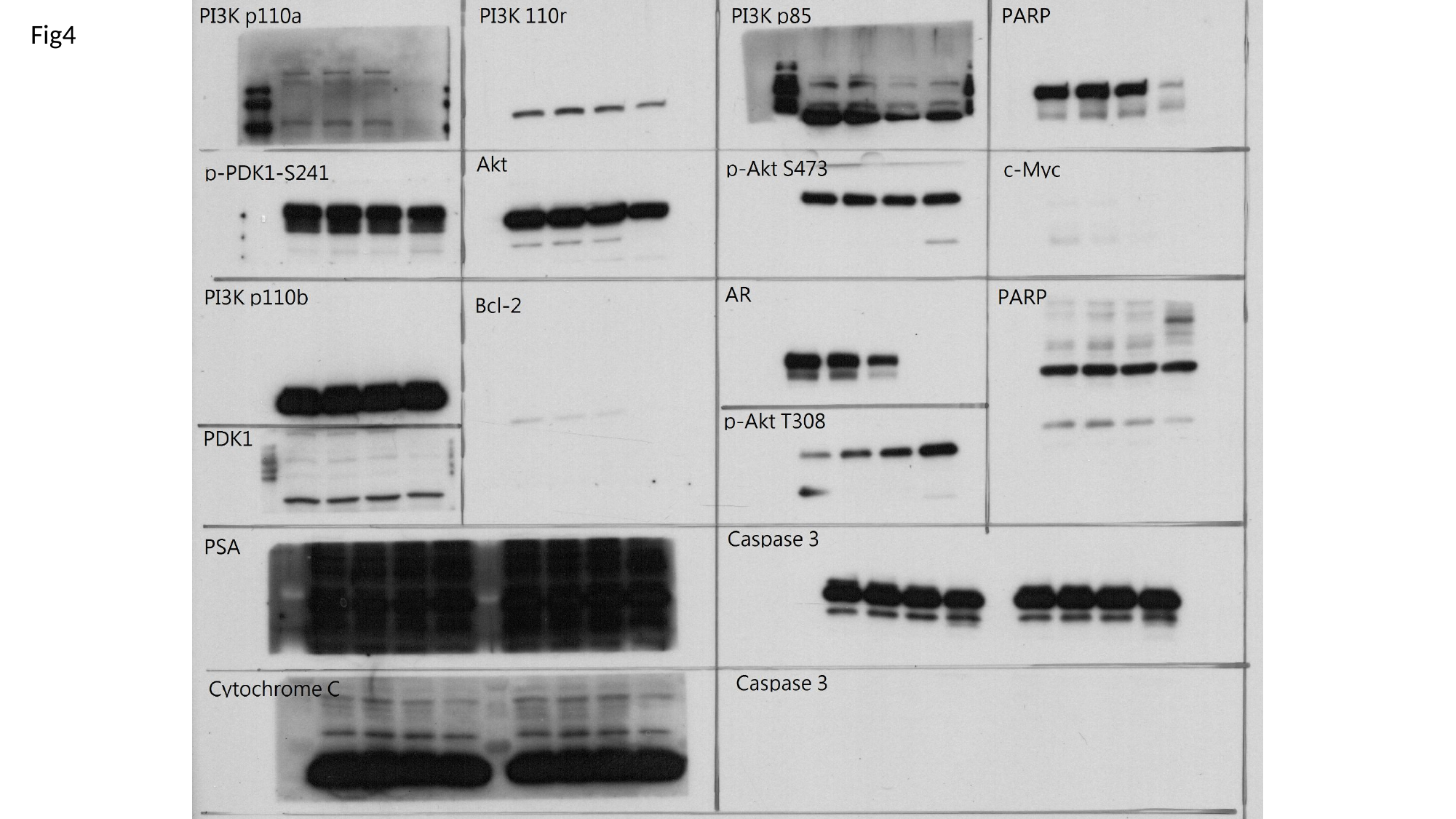

Fig4

## Slide 2
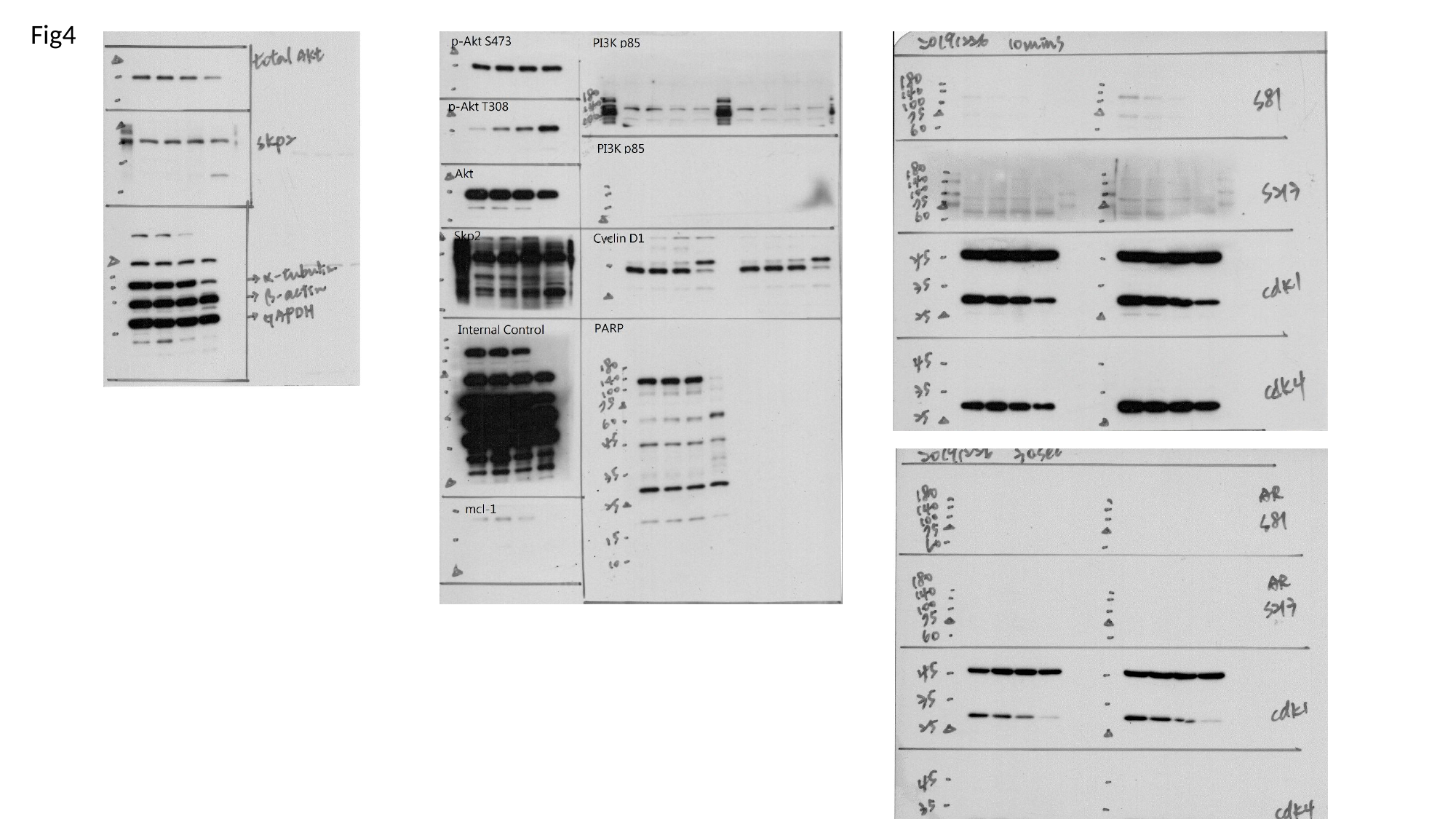

Fig4

## Slide 3
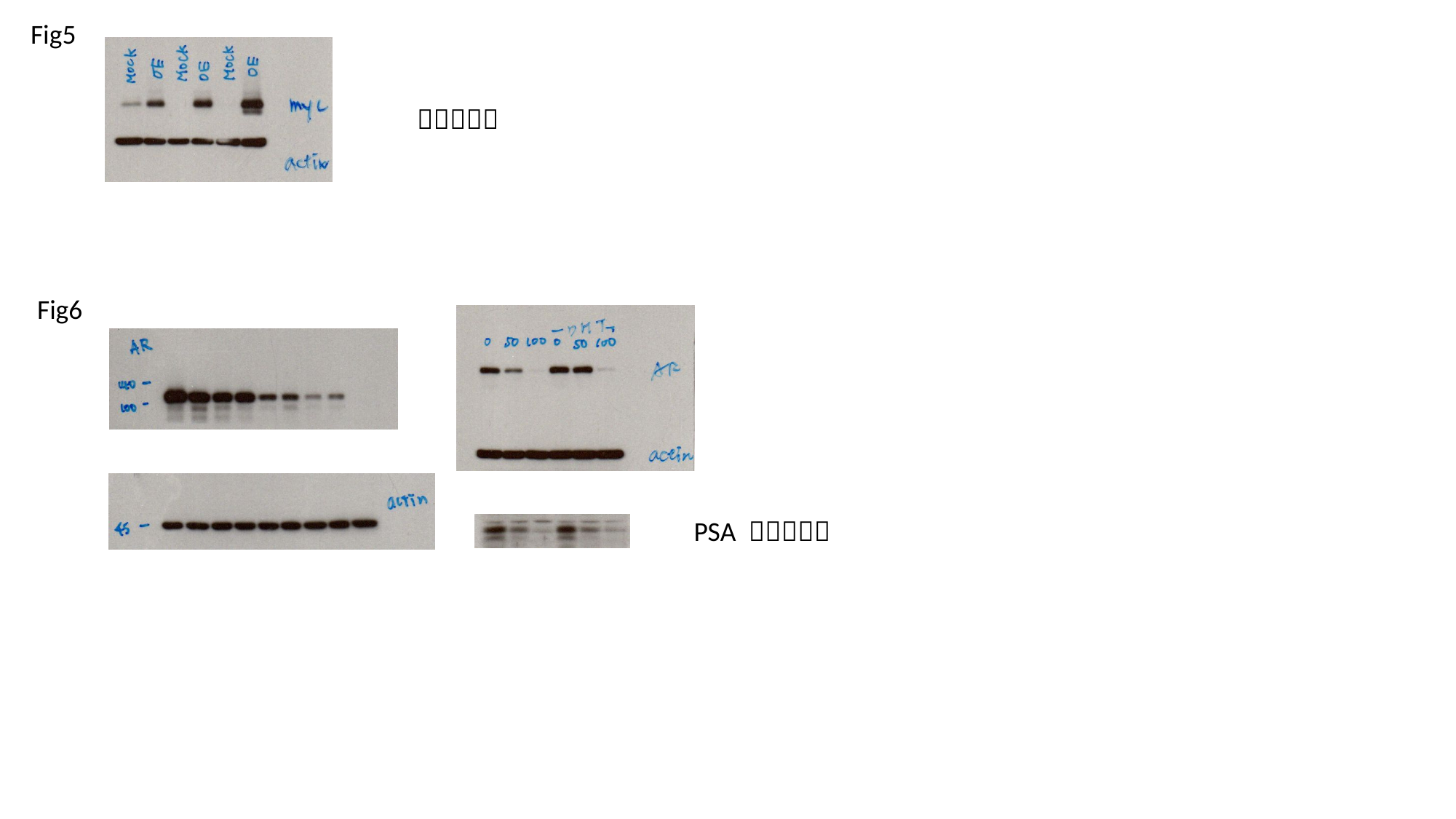

Fig5
截中間那組
Fig6
PSA 未找到底片

Supplement: S1 Raw images — (PPTX) [file pone.0270803.s003.pptx]
