# Supplementary material for: Aspalathus linearis suppresses cell survival and proliferation of enzalutamide-resistant prostate cancer cells via inhibition of c-Myc and stability of androgen receptor
Source: PLoS One. 2022 Jul 1;17(7):e0270803. doi: 10.1371/journal.pone.0270803 (PMC9249401; doi:10.1371/journal.pone.0270803)
Supplement: S1 File — (DOCX) [file pone.0270803.s002.docx]

| Cell culture |  |  |
| --- | --- | --- |
| RPMI-1640 phenol red free medium | HyClone | SH30197.02 |
| FBS | Gibco | 10437-028 |
| P/S | HyClone | SH40003.01 |
| Enzalutamide | MCE | HY-70002 |
| DHEA | Sigma | D4000 |
| DMEM medium | HyClone | SH30243.02 |
|  |  |  |
| **Cell proliferation assay and chemicals** |  |  |
| Hoechst dye 33258 | Sigma | B2883 |
|  |  |  |
| Immunofluorescence staining |  |  |
| goat anti mouse 488 (green) | invitrogen | A11001 |
| goat anti rabbit 594 (red) | invitrogen | A11012 |
|  |  |  |
| **Soft Agar Colony Formation Assay** |  |  |
| low  melting agarose | Lonza | 50101 |
|  |  |  |
| Western blot analysis |  |  |
| ECL | PerkinElmer | NEL105001EA |
|  |  |  |
| Comet assay |  |  |
| Comet assay Kit | CELL BIOLABS | STA-351 |
|  |  |  |
| myc knockdown |  |  |
| ON-TARGETplus Human MYC siRNA-SMARTpool | Dharmacon | L-003282-02 |
| ON-TARGETplus Non-targeting Pool | Dharmacon | D-001810-10 |
| Lipofectamine RNAiMAX Reagent | invitrogen | 13778-150 |
|  |  |  |
| myc overexpression |  |  |
| c-myc plasmid | gifts from Shutsung Liao's lab |  |
| PLNCX2 plasmid |  |  |
| PolyJet Reagent | SignaGen | SL100688 |
|  |  |  |
| **Antibody** |  |  |
| Akt | Cell Signaling | 9272S |
| PI3K p110a | Millipore | 09-481 |
| PI3K p110b | Millipore | 09-482 |
| PI3K p110r | Upstate | 04-402 |
| p-PDK1-S241 | Cell Signaling | 3061S |
| PDK1 | GeneTex | GTX105999 |
| Cyclin D1 | Cell Signaling | 2922S |
| Skp2 | Santa Cruz | sc-7164 |
| GAPDH | Novus | NB300-221 |
| b-actin | Novus | NB600-501 |
| Myc | abcam | ab32072 |
| AR | abcam | ab108341 |
| p-AR-S81 | Millipore | 07-1375 |
| Cdk1 | abcam | ab133327 |
| Bcl-2 | BD | 610539 |
| Caspase 3 | Cell Signaling | 9665S |
| PARP | Cell Signaling | 9542S |
| PSA | Dako | A0562 |
| F-actin | abcam | ab205 |
|  |  |  |
| Cycloheximide | CALBIOCHEM | 239764 |
| 5α-Androstan-17β-ol-3-one (DHT) | Sigma | A8380 |
