# Supplementary material for: Aspalathus linearis suppresses cell survival and proliferation of enzalutamide-resistant prostate cancer cells via inhibition of c-Myc and stability of androgen receptor
Source: PLoS One. 2022 Jul 1;17(7):e0270803. doi: 10.1371/journal.pone.0270803 (PMC9249401; doi:10.1371/journal.pone.0270803)
Supplement: S1 Dataset — (DOCX) [file pone.0270803.s004.docx]

| C4-2 3100r | GRT |  |  |  |  |  |
| --- | --- | --- | --- | --- | --- | --- |
|  | GRT0 | GRT10 | GRT25 | GRT50 | GRT75 | GRT100 |
| 1 | 105.823729 | 113.47712 | 105.625927 | 110.23622 | 76.2296017 | 55.9777854 |
| 2 | 104.043516 | 113.157594 | 104.089163 | 101.958994 | 71.4215071 | 52.5695158 |
| 3 | 110.023204 | 108.501655 | 107.345277 | 99.0832668 | 75.3775343 | 49.4655559 |
| 4 | 99.3267146 | 106.249762 | 104.712998 | 94.8685762 | 71.5888775 | 50.9566739 |
| 5 | 99.6310244 | 101.745977 | 98.778957 | 99.3267146 | 73.2017194 | 50.5002092 |
| 6 | 95.233748 | 97.4856404 | 95.0968086 | 92.4949599 | 69.8543117 | 48.4765491 |
| 7 | 94.6707748 | 101.822055 | 98.809388 | 88.2346228 | 67.6937122 | 44.3531515 |
| 8 | 91.2472897 | 97.1204686 | 92.251512 | 87.1999696 | 55.9473544 | 49.313401 |

96-well Hoechst dye 33258 proliferation assay for C4-2 MDV3100r cells being treated with increasing concentration of GRT for 96h.

96-well Hoechst dye 33258 proliferation assay for PC-3 cells being treated with increasing concentration of GRT for 96h.

PC-3

96-well Hoechst dye 33258 proliferation assay for C4-2 MDV3100r cells being treated with increasing concentration of GRT for 96h

PC-3 cells

| GRT0 | 95.4554 | 94.4613 | 107.3855 | 97.1274 | 99.3267 | 102.9870 | 100.3208 | 109.6450 | 94.6571 | 98.6338 |
| --- | --- | --- | --- | --- | --- | --- | --- | --- | --- | --- |
| GRT10 | 93.5273 | 94.4161 | 94.8981 | 97.3233 | 97.0069 | 97.8655 | 104.5837 | 93.6177 | 100.5468 |  |
| GRT25 | 95.3199 | 96.2839 | 94.4462 | 92.4277 | 95.0939 | 94.8228 | 96.5249 | 98.1216 | 89.8218 | 100.6824 |
| GRT50 | 94.3709 | 93.0001 | 91.4788 | 86.7941 | 93.0303 | 88.0594 | 89.1590 | 87.4569 | 83.8267 | 93.5575 |
| GRT75 | 88.3155 | 83.2392 | 89.0837 | 88.0594 | 84.7756 | 86.8393 | 79.6993 | 85.9204 | 84.5346 | 86.8845 |
| GRT100 | 77.7110 | 73.0263 | 74.7887 | 73.4180 | 72.0472 | 72.0472 | 72.0020 | 72.2732 | 70.5108 | 75.4967 |
| GRT150 | 69.7466 | 64.1081 | 70.8806 | 68.5647 | 72.6585 | 70.2810 | 68.0526 | 71.5106 | 70.5866 | 71.9586 |
| GRT200 | 57.4129 | 57.6788 | 50.9548 | 59.4988 | 52.3538 | 55.1029 | 57.6508 | 55.0889 | 47.2049 | 57.1049 |

96-well Hoechst dye 33258 proliferation assay for C4-2 MDV3100r cells being treated with increasing concentration of GRT for 96h.

C4-2 MDV3100r with or without c-Myc overexpression

| C4-2 MDVr | Mock |  |  |  |  |  |  |
| --- | --- | --- | --- | --- | --- | --- | --- |
| GRT | 1 | 2 | 3 | 4 | 5 | 6 | 7 |
| 0 | 100 | 100 | 100 | 100 | 100 | 100 | 100 |
| 10 | 99.0053421 | 93.0320245 | 95.3984738 | 92.0921429 | 100.898481 | 110.35333 | 102.145152 |
| 25 | 86.7833434 | 77.4873058 | 79.4939469 | 85.3476133 | 97.731411 | 111.338789 | 100.352013 |
| 50 | 69.4226514 | 63.7474235 | 71.2089047 | 67.5422399 | 88.7319781 | 99.0785489 | 89.7845398 |
| 75 | 57.6292496 | 52.134131 | 61.1394802 | 56.2736589 | 65.8592281 | 72.7379341 | 68.1219681 |
| 100 | 53.7460126 | 48.8763763 | 54.6502955 | 49.0721078 | 55.7822155 | 56.6230642 | 58.7129087 |

| C4-2 MDVr | OE |  |  |  |  |  |  |
| --- | --- | --- | --- | --- | --- | --- | --- |
| GRT | 1 | 2 | 3 | 4 | 5 | 6 | 7 |
| 0 | 115.281969 | 125 | 117.012544 | 114.999224 | 115.348371 | 121.615038 | 117.792481 |
| 10 | 110.174568 | 122.17362 | 110.8619 | 103.624425 | 117.289474 | 121.864662 | 119.708271 |
| 25 | 112.403368 | 120.087483 | 104.006012 | 94.4211778 | 114.626566 | 113.870677 | 115.281203 |
| 50 | 98.9237578 | 112.999103 | 98.2715764 | 90.4089758 | 92.3699248 | 94.7578947 | 94.4992481 |
| 75 | 83.0818749 | 96.6464782 | 93.2539453 | 83.9667029 | 69.4887218 | 74.0451128 | 74.3488722 |
| 100 | 97.7771072 | 90.7581875 | 95.4390427 | 85.3937232 | 63.2653061 | 63.0225564 | 62.4691729 |

96-well Hoechst dye 33258 proliferation assay for C4-2 MDV3100r cells being treated with increasing concentration of GRT for 96h.

PC-3 with or without c-Myc knockdown

PC-3 control

| GRT0 | 84.13605 | 101.3852 | 99.64904 | 101.5905 | 103.868 | 103.6253 | 107.1162 | 102.4679 | 96.04615 | 100.1157 |
| --- | --- | --- | --- | --- | --- | --- | --- | --- | --- | --- |
| GRT10 | 86.54421 | 94.23536 | 100.0597 | 91.84588 | 95.8968 | 98.00627 | 96.53151 | 95.33677 | 92.33124 | 97.68892 |
| GRT25 | 103.5133 | 90.63247 | 94.51538 | 87.70161 | 87.88829 | 92.77927 | 94.44071 | 93.35797 | 92.1259 | 95.91547 |
| GRT50 | 94.57139 | 84.13605 | 82.64262 | 80.99985 | 83.20266 | 83.50134 | 84.49074 | 85.70415 | 93.67533 | 98.04361 |
| GRT75 | 79.82378 | 86.30152 | 79.26374 | 77.43429 | 71.16189 | 79.18907 | 82.99731 | 80.58916 | 79.07706 | 87.88829 |
| GRT100 | 84.13605 | 78.62903 | 73.34603 | 73.81272 | 71.60992 | 74.9328 | 75.90352 | 71.29256 | 68.56705 | 79.48775 |

PC-3 c-Myc siRNA

| GRT0 | 90.64144 | 95.88046 | 112.3368 | 110.344 | 105.1693 | 95.71975 | 105.2657 | 101.0231 | 86.52737 | 97.09218 |  |
| --- | --- | --- | --- | --- | --- | --- | --- | --- | --- | --- | --- |
| GRT10 | 95.9126 | 107.7084 | 101.9873 | 121.3684 | 96.97326 | 99.19101 | 96.74827 | 89.00224 | 100.6374 | 86.3988 | 99.59277 |
| GRT25 | 89.38793 | 105.9728 | 100.4124 | 99.06244 | 108.9941 | 105.6835 | 96.00903 | 93.14846 | 96.16973 | 85.72384 | 98.05642 |
| GRT50 | 82.34901 | 85.27386 | 87.23447 | 89.93434 | 82.70256 | 93.05203 | 74.66726 | 80.38839 | 73.83159 | 82.86327 | 83.22968 |
| GRT75 | 73.28519 | 76.37074 | 78.3635 | 91.18784 | 74.6994 | 68.59257 | 67.53191 | 70.03892 | 72.70664 | 68.52829 | 74.1305 |
| GRT100 | 69.65323 | 60.30013 | 68.94612 | 69.5568 | 66.47125 | 62.0679 | 57.27886 | 61.42508 | 61.93934 | 59.72159 | 63.73603 |
