# Supplementary material for: Aspalathus linearis suppresses cell survival and proliferation of enzalutamide-resistant prostate cancer cells via inhibition of c-Myc and stability of androgen receptor
Source: PLoS One. 2022 Jul 1;17(7):e0270803. doi: 10.1371/journal.pone.0270803 (PMC9249401; doi:10.1371/journal.pone.0270803)
Supplement: Attachment — Submitted filename: Rebuttal letter PLOS One 20220525.docx [file pone.0270803.s005.docx]

05/26/2022

Dear Editor,

We are here re-submitting the paper “Aspalathus linearis suppresses survival and proliferation of enzalutamide-resistant prostate cancer cells via inhibition of c-Myc and stability of androgen receptor” by Bi-Juan Wang and Shih-Han Huang et al. for consideration for publication as research paper. No related content of this research has been submitted or published elsewhere. We have revised the manuscript and added two experiments according to the suggestions made by the reviewer, as well as answered all the questions raised by the reviewers. We also included our original Western blot scans as well as provide a file of minimal data set as supplemental material for review purpose. We have revised the manuscript to meet PLOS ONE's style requirements. The pharmaceutical-grade green rooibos extract (GRT, containing 12.78% aspalathin) being used in this study was extracted and produce by the co-author Prof. Christo J.F. Muller from Medical Research Council of South Africa. We included the HPLC analysis of this batch of GRT as supplemental figure. We have corrected the funding information in the submission system so that the information is now consistent with that in the manuscript. We hope that the current revised manuscript fulfill the requirement of PLOS One.

The following is a response to the reviewers’ concerns and questions.

**Reviewer #1:** The manuscript by Huang et describes the growth inhibitory effect of green rooibos extract on the proliferation of the castration resistant C4-2 prostate cancer cells, a derivative which is Enza resistant and the androgen receptor negative expressing PC3 cell line. Authors continue with the androgen receptor positive C4-2 cells and reveal that the extract induces apoptosis. Interestingly, some factors are reduced in protein level including c-Myc. Overexpression of c-Myc reduced extract-mediated growth inhibition. This is a continuation of experiments of the group previously revealed that the rooibos extract inhibits castration resistance prostate cancer cells through inhibition of YAP and AKT signaling.

Therefore, some additional novelties and specificity must be added to the manuscript

**Answer:** We thank reviewer for the affirmative comment.

**Major points:**

1. Authors show reduction of cell viability by the extract in prostate cancer cell lines. This may also indicate an unspecific effect by using 100microgram per ml. Authors should reveal a cell specific response by treatment with a primary explant or primary, non-immortalized, human cells with the same culture condition that is resistant to the treatment. The general question is whether these high doses will lead to toxicity in non-cancerous cells?

Answer: We thank the reviewer for raising this important question. We now included the data of HUVEC (human umbilical vein endothelial cells) and HEK293 (human embryonic kidney cells 293). The HUVEC cells are normal human primary cells and HEK293 cells is an established cell line with mutant version of SV40 large T antigen. Proliferation of HUVEC cells was not affected by 10-75 μg/ml GRT and was relatively resistant to 100 μg/ml GRT, while HEK293 was not affected by GRT at all. This observation suggested that enzalutamide-resistant PCa cells were more sensitive to GRT treatment than non-cancerous human cell lines.

2. Further, the data suggest that those cell lines with c-Myc overexpression are resistant to the treatment. Meaning that those cancer cells, which represent the more aggressive types, which overexpress c-Myc, are treatment resistant. Please discuss this issue.

Answer: We thank the reviewer for raising this very interesting question. We performed a new Western blotting assay to compare the protein expression of c-Myc in C4-2 MDV3100r and PC-3 cells being treated with 0 or 100 μg/ml GRT. PC-3, which was more resistant to GRT treatment, expressed higher level of c-Myc protein as compared to C4-2 MDV3100r (new Fig. 6C). We also added a new c-Myc knockdown experiment on PC-3 cells to examine if knockdown of c-Myc increases the sensitivity of PC-3 cells to GRT treatment. Knockdown of c-Myc with siRNA in PC-3 cells increased the sensitivity of PC-3 cells to GRT treatment (new Fig. 6A, 6B). We added the following discussion in the Discussion, “Our study suggested that GRT treatment significantly repressed the protein abundance of c-Myc in enzalutamide-resistant PCa cells. We observed that overexpression of c-Myc enhanced the resistance of C4-2 MDV3100r cells under GRT treatment while knockdown of c-Myc in PC-3 increased the sensitivity of PC-3 cells to GRT treatment. We also observed that level of c-Myc protein expression correlated to resistance of PCa cells to GRT treatment. These findings suggested that c-Myc is one of the main targets of GRT treatment in enzalutamide-resistant PCa cells. The expression level of c-Myc in enzalutamide-resistant prostate tumors may vary in different patients and we predict that those enzalutamide-resistant prostate tumors with lower expression level of c-Myc will be more sensitive to GRT treatment. We also predict that combined treatment of GRT with pharmaceutical inhibitor targeting c-Myc will enhance the regression of tumors.”

**3. Unclear is why authors focus on androgen receptor degradation, while they reveal that the androgen receptor-negative expressing PC3 cell line is also inhibited in growth by the extract. It would be more helpful to make a general statement to overexpress c-Myc in PC3 cells to reveal whether the rooibos extract targets c-Myc also in these cell lines and whether c-Myc overexpression can ameliorate the apoptotic effect. Thus, reveal in PC3 cells whether rooibos extract targets c-Myc downregulation and by overexpression whether c-Myc is the key factor for rooibos-mediated growth inhibition.**

Answer: We thank the reviewer for raising this important question. A majority portion of CRPC tumors still express AR. We therefore examine if GRT may suppress the protein expression, phosphorylation, signaling transduction, and stability of AR in the AR-positive C4-2 MDV3100r cells. However, as the reviewer pointed out, PC-3 cells are AR-negative PCa cells. As PC-3 cells express much higher c-Myc protein as compared to C4-2 MDV3100r cells (new Fig. 6C), we chose to knock down c-Myc in PC-3 cells and to overexpress c-Myc in C4-2 MDV3100r cells to determine if c-Myc is one of the main targets of GRT in CRPC cells. We observed that overexpression of c-Myc enhanced the resistance of C4-2 MDV3100r cells under GRT treatment while knockdown of c-Myc in PC-3 increased the sensitivity of PC-3 cells to GRT treatment (new Fig. 6A, 6B). These observations suggested that c-Myc is one of the main targets of GRT in enzalutamide-resistant PCa cells.

**Error bars in Fig. 5B with no GRT are missing.**

Answer: We thank the reviewer for noticing this mistake. We now added the error bars into the condition with GRT treatment in Fig. 5B.

**Label “Actin” with a capital “A”.**

Answer: We thank the reviewer for noticing this mistake. We now corrected all “actin” to “Actin”.

**Reviewer #2: Enzalutamide is commonly used in treating prostate cancer (PCa) patients, but frequently results in resistance in PCa patients. In this manuscript, the authors examined the effect of a pharmaceutical-grade green rooibos extract (GRT, with 12.8% aspalathin) on the suppression of the survival and proliferation of enzalutamide-resistant prostate cancer (PCa) cells as well as the mechanism. By using two enzalutamide-resistant prostate cancer cell lines C4-2 MDV 3100r and PC-3, they demonstrated that both are resistant to 10uM MDV compared to the C4-2 cells, and identified the IC50 concentrations of GRT in C4-2 MDV 3100r and PC-3 both cell lines. Furthermore, GRT treatment in a dose-dependent manner can reduce cell proliferation, decrease colony formation in soft agar and induce apoptosis by comet assay in C4-2 MDV 3100r cells. In the mechanism study by examining a panel of signaling proteins in cell cycle, apoptosis, cell survival, and androgen receptor (AR) signaling in C4-2 MDV 3100r treated with different concentrations of GRT, the authors found the increasing dose of GRT reduces the levels of the proteins in cell cycle and cell survival as well as increases the proteins in apoptosis. In addition, they indicated that Myc overexpression elevates the resistance in C4-2 MDV 3100r cells under GRT treatments. Moreover, they showed that GRT treatment diminish AR expression, stability, and PSA expression in C4-2 MDV 3100r cells. The authors suggest that GRT may serve as a potential adjuvant therapeutic agent for enzalutamide-resistant prostate cancer. Overall, this is a nice and thorough work providing the mechanism study of GRT on enzalutamide-resistant prostate cancer cells.**

**Answer:** We thank reviewer for the affirmative comment.

**However, there are some typos or grammatical errors as below need to be corrected.**

**1. In line 56, 280, and 347, it should be “Interestingly” not “Interesting”.**

**2. In line 78, delete “in” before every year.**

**3. Line 82, delete “will”.**

**4. Line 85, should be “patients”.**

**5. Line 108, play “an” essential role.**

**6. All the antibodies and special reagents or kits should be included with the catalogue number in the Materials and Methods.**

**7. The first paragraph in the first section of the results from line 208 to 217 can be moved to the Materials and Methods.**

**8. The more correct way of labeling should be “phospho-PDK1 (S241)” in line 253 and 308, and 427, similarly like “phosphor-AR (S81)” in line 254 and 428.**

**9. Line 276, “reduces”.**

**10. Line 292, “mediated”.**

**11. Line 432, “enhanced”.**

Answer: We sincerely apologize for these typos in the manuscript and we appreciate the reviewer for pointing out these typos. We have now corrected all these typos.

**In addition, the images of the figure 1D, 1E, 2C, and 6A should include the scale bar.**

Answer: We thank the reviewer for finding these mistakes. We have now added scale bars into these figures.

Sincerely,

Chih-Pin Chuu, Ph.D., Associate Investigator/Associate Professor

Institute of Cellular and System Medicine, National Health Research Institutes, Taiwan
